# Barriers and Facilitators of Participation in Sports: A Qualitative Study on Dutch Individuals with Lower Limb Amputation

**DOI:** 10.1371/journal.pone.0059881

**Published:** 2013-03-22

**Authors:** Mihai Bragaru, C. P. van Wilgen, Jan H. B. Geertzen, Suzette G. J. B. Ruijs, Pieter U. Dijkstra, Rienk Dekker

**Affiliations:** 1 Department of Rehabilitation Medicine, Center for Rehabilitation, University Medical Center Groningen, University of Groningen, Groningen, The Netherlands; 2 Faculty of Human Movement Sciences, University of Groningen, Groningen, The Netherlands; 3 Transcare, Trans-disciplinary Pain Management Centre, Groningen, The Netherlands; 4 Center for Sports Medicine, University Medical Center Groningen, University of Groningen, Groningen, The Netherlands; University of Rome, Italy

## Abstract

**Introduction:**

Although individuals with lower limb amputation may benefit from participation in sports, less than 40% do so.

**Aim:**

To identify the barriers and facilitators that influence participation in sports for individuals with lower limb amputation.

**Design:**

Qualitative study.

**Participants:**

Twenty six individuals with lower limb amputation, all originating from the Dutch provinces of Groningen and Drenthe, of which 13 athletes.

**Methods:**

Semi-structured interviews were used to gather information. Following thematic analysis, emerging themes were organized in three categories Technical, Social and Personal.

**Results:**

Sport was perceived as enjoyable activity that would help participants to become and stay healthy, improve the number of social contacts, reduce phantom pain and decrease daily tension. Inadequate facilities, problematic transportation, trivialization from others, poor health and lack of motivation or the lack of a sports partner were barriers commonly mentioned by non-athletes. Remarkably, while all athletes were successful prosthetic users, the majority chose to participate in sports for which prosthesis was neither required nor needed.

**Conclusions:**

Each individual with lower limb amputation needs to be counselled according to the barriers and facilitators he/she personally experiences. Athletes appeared to be more proactive in searching for a solution and also appeared less discouraged by failing.

## Introduction

According to the general perception, regular participation in sports or physical activities (PA) is considered a fundamental element of a healthy life style. Literature also supports this general opinion by presenting the numerous benefits regular participation in sports or PA has on reducing type 2 diabetes and improving cardio-vascular function [1], physical functioning [2], social environment and the psychological traits [3]. Several reviews showed that regular participation in sports or PA has at least the same positive influence on the individuals with a physical disability as for the able bodied ones [4]–[6]. Amputation of a limb is a physical disability that appears to have a significant negative impact on physical and psychosocial functioning [7], [8]. Regular participation in sports or PA improves the physical [9]–[11] and psycho-social [12], [13] functioning of individuals with lower limb amputation (LLA), thereby decreasing to some degree the burden of amputation [14].

The participation rate in sports or recreational PA for individuals with LLA ranges from 11% to 60% [14].For example in the Netherlands, between 32 and 39% of individuals with LLA participate in sports [15], [16]. Participation in sports of individuals with LLA was negatively associated with various factors, such as older age, vascular cause of amputation, a more proximal level of amputation and the fact that the individual did not participated in sports before the amputation [14]. Although these factors may be used to predict the likelihood of participation in sports for an individual with LLA based on his or her personal characteristics, these factors do not explain why only a third of the Dutch individuals with LLA participate in sports [15], [16] while around 56% of the general Dutch individuals participate in sports [17].

Participation in sports and/or PA of able-bodied individuals is influenced by various factors, such as socioeconomic status, presence of a sports partner, education, the amount of free time, age and health status [18], [19]. Some may suppose that the above mentioned factors may also influence the participation in sports or PA of individuals with LLA. Nevertheless, individuals with LLA differ from the general population in terms of physical and psycho-social functioning [7], [20], [21]. Factors related to the amputation itself are expected to influence participation in sports for individuals with LLA. Therefore, it is important to address individuals with LLA as a separate group with specific requirements, needs and experiences. For example, it was identified that through regular participation in sports individuals with LLA increase their number of social contacts [12], have a better self-esteem [22] and a better body-image of themselves [23]. Unfortunately these factors were only associated with participation in sports or PA, while the causality of the relation was not thoroughly investigated.

In the last decade, regular participation in sports or PA has become widely advocated through various media channels as well as by various health professionals [24]. Unfortunately, still a large percentage of the general population does not participate regularly in sports or PA [25]. The situation is similar also for individuals with physical disabilities, including individuals with LLA. There is the general opinion that the percentage of individuals with physical disabilities that participate in sports has to increase in the coming years [26], [27]. Identifying the barriers for sports participation of individuals with LLA may offer an explanation of the low participation rate recorded by the literature [4], [14], [26], [27]. In addition, identifying the facilitators of regular participation in sports may lead to the development of better strategies aimed to increase participation in sports of those individuals. Consequently, the aim of this study was to identify the barriers and facilitators that influence participation in sports for individuals with LLA. With regards to the status of sports participation, an individual with LLA will either participate in sports (athlete) or not participate in sports (non-athlete). In order to get an overview of the barriers and facilitators that influence sports participation of individuals with LLA one should address both athletes and non-athletes alike. In this manner the barriers experienced by non-athletes as well as the possible facilitators for sports will become clear and a specific plan of action may be developed. When developing this action plan, the facilitators (motivators) experienced by athletes as well as their strategies to overcome various barriers to sports participation may be useful.

Participation in sports represents a human behaviour and as any human behaviour is a complex cognitive process which implies decision-making based on the assessment of various factors related to personality, beliefs, attitudes, personal goals, social norms and environment [28]. Qualitative research methods focus “*more on the (whole) person in his/her life world, relying more on subjective reports and experiences, giving more room for meaning of life, allowing for more openness for unanticipated meanings and connections…*” [29]. Additionally, focusing on the individual allows him to express his own feelings and personal experiences, thus “giving him voice” [30]. Depending on the methods used for gathering and analysing data there can be three major types of qualitative research Ethnography, Grounded Theory and Phenomenology [31]. Ethnography is most commonly used in anthropology and is characterized by using ethnographic data sources like stories, legends or even the general perceptions of a group. Grounded Theory aims to develop a theory about the phenomena of interest by coding and analysing the data and later organizing the emerging factors into categories. Phenomenology aims to describe individual experiences and behaviour and is preferred when there is little known about the subject of research and the researcher aims to acquire a broad and a complete set of data. Considering that the aim of the current study is to identify personal barriers and facilitators that influence participation in sports of individuals with LLA a Phenomenological approach will be ideal.

## Methods

### Ethics Statement

The medical ethical committee of the University Medical Center Groningen was informed on the exact research methodology of this study and it judged that no specific approval was needed for this study (M10.085238). Participants who agreed to be interviewed were asked to sign the informed consent and return it to the sender along with their current status of participation in sports and contact details. All the interviewed participants signed the informed consent form.

### Data Collection

Personal semi-structured interviews were held to capture both the interviewee’s opinion and to gather a sufficient and broad amount of information. The interviews were conducted in Dutch by two people: SR was the interviewer (Dutch native speaker), and MB was the observer (conversationally proficient in Dutch). The observer assessed non-verbal reactions and verified the topics discussed. The interview took place at the participant’s home to provide a relaxed environment. Interviews were recorded on minidiscs (MD®) and transcribed verbatim by SR. Prior to this study, SR received interview training, and the interview guide was piloted three times. The first two pilots were performed with one of the members of the research project (RD) playing the role of an individual with LLA, while the third and final pilot was performed with an individual who had a LLA. The three tests were not used in the analysis. Following each test, the interview guide was adapted and improved in order to be able to record at its best interviewee’s meanings. The last version of the interview guide was applied in all interviews.

The interview started with informal conversation aimed at relaxing the interviewee and creating a venue for discussion. This conversation was also used also to inform the interviewee about the aim of the project and to present an overview of the interview. Thereafter, the interviewee was asked if he/she had any questions, and if he/she agreed to proceed. First, personal characteristics, such as age, gender, education level, and comorbidities, and amputation characteristics, such as level and cause, were asked for. Next, the interviewee was invited to speak freely about why he or she did or did not participate in sports. When short answers were provided, interviewees were invited to explain their answer in greater detail. If the conversation deviated from the topic or the interviewee centred on one specific topic only, the interviewer used the interview guide to start a new topic of discussion. The questions contained by the interview-guide (Appendix S1) were all open-ended and related to 1) personal characteristics such as attitudes toward sport, self-efficacy or past behaviour; and 2) social and technical environment. Additionally, factors identified by means of a systematic review [14], including age, gender, civil status, education level, employment status, amputation’s level, aetiology and date, health status, prosthesis, access to sports facilities, information, time, pain, fear, shame, dependence on others, previous experience with sports, costs, and pleasure from sports, were organized into a list that was to be assessed at the end of the interview as a consistency check or to be utilized if the interview grew stagnant [32]. At the end of the interview, SR asked the observer if any topics require further probing.

### Participants

Inclusion criteria for participants were: a) 18 years of age or older; b) a minimum of 12 months since the amputation; c) LLA more proximal than the ankle; d) able to speak and understand Dutch. Participants were organized in two groups: individuals who participated in sports (athletes) and individuals who did not participate in sports (non-athletes). In order to be able to distinguish athletes from non-athletes, sport was defined as “*an activity involving physical exertion, with or without game or competition elements, with a minimal duration of half an hour per time and a minimal duration of 60 minutes per week and where skills and physical endurance are either required or to be improved”* [33]. A total of 47 individuals with LLA agreed to participate in the study, of which 26 were interviewed.

### Sampling

According to purposeful sampling, participants were recruited from a group of individuals with physical disabilities who regularly participated in sports organized by a rehabilitation centre and a prosthetic manufacturer located in one of the Northern provinces of the Netherlands. During a group meeting, the individuals with physical disabilities were informed about the purpose of the study, the interview and the possible burden associated with it and data confidentiality. Individuals fulfilling inclusion criteria were invited to participate in the study by either SR or MB. The interview was scheduled after written informed consent was given. Additional participants were recruited through a prosthetic manufacturer who sent an invitation letter and a form for informed consent to every individual in their database who fulfilled the inclusion criteria. The letter contained information identical to the one presented to the participants of the sports group.

Participants recruited through the prosthetic manufacturer were contacted in two rounds. Initially, 87 individuals with LLA were invited to participate, 17 of whom (7 athletes) agreed to participate. One of the individuals with LLA who agreed to participate could not be contacted. The remaining 16 individuals with LLA and 2 others recruited from the group of individuals with physical disabilities were interviewed including 9 athletes. After these interviews data saturation was not reached. Consequently, a second round of interviews was scheduled, and invitations were sent to 147 participants recruited through the same prosthetic manufacturer, of which 28 (17 athletes) agreed to participate. Sampling continued until data saturation was reached. Interviewees were randomly selected from the pool of remaining participants. Characteristics of the 26 interviewees are summarized in Table 1. Athletes were on average younger (49.9±15.7 years) and had less vascular amputations (38.5%) as compared to non-athletes (64.6±7,89 years) respectively (77%).

**Table 1:** Participants characteristics.

Code	Gender	Age	Level of education	Level of amputation	Years since amputation	Cause of amputation
NA1	man	76	High	TT	20	Vascular
NA2	man	59	Low	TF	8	Trauma
NA3	man	72	Low	KD	7	Vascular
NA4	man	59	High	KD	16	Trauma
NA5	man	64	Low	TT	6	Vascular
NA6	man	72	High	TT; TF	10	Vascular
NA7	man	73	Low	TF	2	Vascular
NA8	man	64	Low	TT	10	Vascular
NA9	woman	61	Low	TF	9	Oncologic
NA10	man	67	Average	AD	30	Vascular
NA11	woman	49	High	HD	4	Vascular
NA12	woman	55	Low	KD	8	Vascular
NA13	man	69	Low	KD	14	Vascular
A1	man	53	High	KD	10	Vascular
A2	man	63	High	TT	6	Trauma
A3	man	50	Average	TT	35	Trauma
A4	woman	77	Low	TT	2	Vascular
A5	woman	21	Average	TF	7	Oncologic
A6	man	30	Average	KD	6	Vascular
A7	woman	48	Average	TT	3	Vascular
A8	man	51	High	HD	7	Oncologic
A9	man	44	High	TF	19	Oncologic
A10	man	63	Low	TT;KD	12	Trauma
A11	woman	36	Average	TF	15	Trauma
A12	man	69	Low	TT	5	Vascular
A13	man	44	High	TT	14	Trauma

Legend: NA- non athletes; A- athletes; high- university or college equivalent; average- vocational training; low- primary school or high school; AD- Ankle disarticulation; TT- transtibial amputation; KD- knee disarticulation, TF – transfemoral amputation; HD – hip disarticulation.

All participants in the study received a flower bouquet of symbolic value (10 euro). The individuals who wanted to participate but were not interviewed were contacted and told that data saturation had been reached and therefore they would not be interviewed. These individuals all received a check by mail (10 euro).

### Data Analysis

Immediately after the interview, the name of the participant was replaced with a code representing the level of sports participation and the interview number. For example, the first athlete interviewed received the code A1, whereas the first non-athlete received the code NA1. Data analysis was intertwined with the interview process from the beginning. This analysis helped the interview process, provided new topics and enabled detection of data saturation. Data saturation, meaning that no new codes emerged from the analysis, was reached after 24 interviews. Two additional interviews were performed in which data saturation was confirmed. Because we were undertaking the first qualitative study aimed at identifying both barriers and facilitators of participation in sports for a individuals with LLA, thematic data analysis was conducted: 1) data familiarization; 2) generating initial codes; 3) searching for themes; 4) reviewing themes; 5) defining and naming themes; 6) producing the report [34]. ATLAS.ti® computer software was used to facilitate organization of the data and emerging factors into themes and categories of themes and to visualize the relationship between these.

Prior to data analysis, SR and MB developed a codebook based on the available literature. During the preliminary assessment, several inductive and open codes were added to the codebook. Data were coded by SR using the codes already existent in the codebook. Along the way, emerging new codes were also added to the codebook. After coding the 26 interviews, the codebook contained all of the identified deductive, inductive and open codes. To check coding consistency, PvW independently coded 10 randomly selected interviews. The differences in coding were discussed until an agreement was reached. The resulting codebook and coding strategy were considered definitive. For the final step, MB checked for consistency and validity of the coding using the final version of the codebook. In case of coding inconsistency a third person was asked to give a binding verdict. Similar codes were grouped together and formed a factor. Later, similar factors were grouped into themes and, finally, into 3 categories: technical, personal and social. The factors, themes and categories were developed by MB in consensus with SR. The final construct was presented during a group meeting to the entire research group. The quotes were translated into English by a native Dutch speaker who took into consideration regional characteristics and idioms. To ensure the accuracy of the translation, a second native Dutch speaker was asked to translate a sample of randomly selected quotes from English to Dutch. The two versions of the same quote were compared for consistency, and a final version was chosen.

## Results

**Figure 1:**
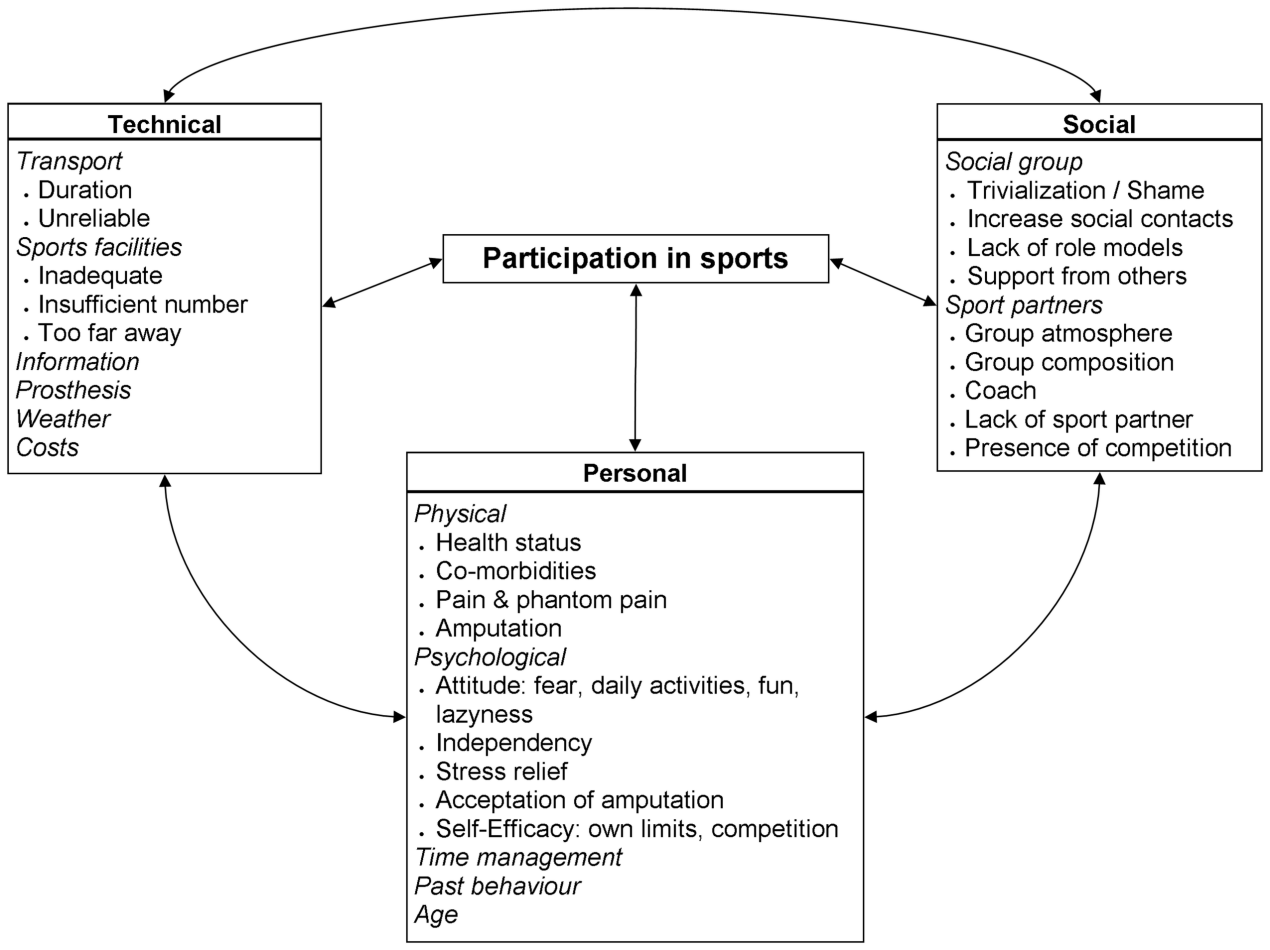
The 3 categories that summarize the factors and themes that influence participation in sports for individuals with LLA. Categories are presented in *bold*, themes are *italics* and factors are in plain text.

The identified factors emerging from the interviews were organized into specific themes and consequently into bigger and broader 3 categories (fig. 1).

### Barriers

A number of factors, such as older age, poor weather or high cost, were negatively associated with participation in sports by several interviewees. We decided not to address these factors in the results because they are not specific to our population. Instead we focused on the factors which are either specific to our population or appeared most frequently in the interview.

#### Technical

Technical barriers include factors and themes related to transportation, infrastructure (sports facilities), information and prosthesis.

#### Transportation

In general, individuals with LLA use either their own vehicles, or a bus or taxi (covered by their health insurance) to travel to and from sports facilities. A barrier mentioned by athletes and non-athletes alike was their dependency on a bus or taxi. The general opinion was that it either takes too long to reach the destination or that the transportation is unreliable. *“That is also unpleasant and tiring <*going to the sport school*> with the taxi….Once I’ve been waiting for 3 hrs. I don’t want that again”* (NA7).

#### Sports facilities

Sports facilities were generally perceived as minimal and not well-adapted to the needs of individuals with LLA. Additionally, the availability of sports facilities was generally perceived as a barrier. Non-athletes mentioned that they *“…would prefer to go to a sports facility in their neighbourhood.”* (NA11). Unfortunately, there were insufficient sports facilities in close proximity to their homes, and this condition was unsatisfying. Athletes also mentioned that *“if a regular sports school would have better access for wheelchair users then they would have chosen for a regular one”* (A6).

#### Prosthesis

The majority of non-athletes mentioned that their prosthesis may be a potential barrier to their participation in sports. *“I can’t walk further than 200–300 m and afterwards that thing* <prosthesis> *begins to cause corns or blisters, thus I have to stop.”* (NA10). When the interviewee was asked if a better prosthesis would help him to exercise more, the answer was *“No, because I have the best there is.”* (NA10). Thus, it appears that the prosthesis had no influence whatsoever on his participation in sports. A number of athletes felt that their prosthesis was a hindrance when participating in sports or was unnecessary, and therefore, chose to take part in wheelchair sports or another type of sports in which the prosthesis was not required. *“As a matter of fact, I feel better if I participate in sports without my prosthesis…I actually find it more comfortable, <*because*> the prosthesis just feels like a block on your leg…is not actually yours. If I participate in sports without the prosthesis I’m more relaxed, I don’t have to think about it. <*prosthesis*>”* (A5). Overall, the prosthesis was not perceived to be a barrier for participation in sports. Athletes for whom the prosthesis represented a barrier for sports proactively searched for a solution to their problem *“with my previous prosthesis I didn’t dare to get into the water….so I actively requested that my following prosthesis would allow me to use it in water, even in salt water.”* (A2).

#### Social

Social barriers include factors and themes related not only to the interactions of individuals with LLA with their social groups or sports partners but also to the perceived lack of support they received from their social groups.

#### Social group

The social group includes the individuals with whom the interviewee interacts on a regular or irregular basis, such as friends, family or other individuals, on the sports field or at the gym. Shame and support are the main factors in this theme. Sometimes, able-bodied individuals stare at the individual with LLA or even refuse to attend the same sports centre. This behaviour generates a state of discomfort and may have a negative impact on participation in sports, as one individual with LLA mentions: *“…some things you have to accept, however it may be…but yeah, the people who went to that gym, they did not accept me. Some people stopped attending* <the same gym>, *because of me. Yes, that was unpleasant for me but also for the people. And afterwards I had to make a choice. And my choice was, that I don’t want to sport in that group anymore….Afterwards I tried in another place, but it was exactly the same, people can’t accept it <*interviewee starts to cry*>.”* (NA12). These negative experiences were not limited only to the non-athletes group with some of the athletes sharing similar experiences *“People do not seek contact by a normal sports school, they just stare in a weird way at you, but they will never come to you and ask what is wrong with you. Then you feel looked at in a weird way.”* (A5).

#### Sports partners

Negative interactions with the team members or the coach may influence sports participation in athletes and non-athletes alike. Lack of a sports partner was viewed by non-athletes as a major barrier. *“I think that this* <alone> *is the reason…I don’t like this at all…” (*NA5). Additionally, some non-athletes and athletes alike also mentioned that they would not like to be in the same group as other physically disabled individuals, *“…and I don’t have to sit between disabled…it is so annoying and unpleasant, I go sick from it.”* (NA9) *or “I do it* <sport>*preferably together with normal individuals than with handicapped ones. It does not appeal to me to be part of that group.”*(A3).

#### Personal

Personal barriers include factors and themes related to physical health or psychological attributes of individuals with LLA. In addition, past experience, time management and age were assigned to this category.

#### Physical

Current health status, medication and pain were frequently adressed in this theme. Both athletes and non-athletes stated that if they have a stump wound, other problems with their stump or any other serious health problems they would end their participation in sports, temporarily or indefinitely. For some interviewees, pain, whether from a stump or phantom, acted as a barrier. *“Because I have a low pain threshold, I can’t participate in sports adequately”* (NA8).

#### Psychological

Feelings, thoughts and perceived barriers were included in this theme. Interviewees’ thoughts about what others may think, acceptance, self-efficacy and their feelings and core beliefs are some examples of these factors.

Confrontation with their own limits or with other obstacles that they were unable to overcome was a barrier for some. This confrontation may be experienced when comparing their capabilities prior to the amputation or by comparing themselves to other individuals who have different performance levels. *“Now, if I swim, the speed is gone and you always have a disadvantage… swimming is not what it used to be, all elderly swim faster than me……I stopped with it…” (*NA4). Even if they do not feel physically disabled, asking for help from others, or feeling dependent on others, is unacceptable for most of the individuals. *“You always need help*<when participating in sports>*…That’s a disability….Now, I don’t feel disabled, I can do everything…”* (NA13) or *“If others have to help me, then you still get sometimes an unpleasant feeling.”* (A9).

Sometimes even the thought of becoming injured acted as a barrier. *“If I ever fall again on a tile, stone floor or whatever, then I know that I will break my hip…”* (NA10). Several of the non-athletes had the impression that they obtained enough PA during their daily activities and that therefore they did not need to participate in sports. *“I do my own household …the 30 minutes physical activity per day I get easily.”* (NA9). They also mentioned that their core beliefs can be a major barrier for participation in sports. Common factors depicting their core beliefs were, for example, a lack of interest in sports, not being in the right mood for sports or just laziness: *“I’m too easy and I think also that I’m too lazy by nature…”* (NA4).

#### Past behaviour

Participation in sports prior to the amputation was never mentioned as a major barrier for participation in sports following the amputation. Past participation was usually mentioned in association with another “free quoted” factor, such as, *“I wasn’t an athlete before the amputation and afterwards, also due to my amputation, I did not become one…”* (NA2). Regardless of the association with other factors, most of the non-athletes mentioned that they were also inactive prior to the amputation.

#### Time management

A busy schedule or a busy daily life can be a barrier. In general, taking care of children, daily household activities or work were responsible for decreasing the amount of time available for sports. *“Time has some influence, I have to take care of my household, thus you get less and less time to do something else* <sport>…*”* (NA10).

### Facilitators

#### Technical

Factors and themes related to information and the assistive devices used during sports were included in this category.

#### Information

Being advised by their attending physician or general practitioner is a motivation to start participating in sports. The vast majority of interviewees remembered receiving information about sports, either during their rehabilitation or in the period closely following it. *“In the rehabilitation center, immediately following the amputation, we had to participate in wheelchair sports. In this way you see what you can do.”* (A5) Even so, some of the non-athletes were not motivated by this to start participating in sports *“Yes, that was good* <receiving information>. *The only thing is that I never used that information.”* (NA1).

#### Prosthesis

The prosthesis was not viewed as a direct motivator for sports but as an indirect one. For example, athletes stated that participating in sports would help them to make the best use of their prosthesis. *“If I keep my body in a good condition …then I can walk for a full day on my prosthesis. Thus, if I’m more active, I can use my prosthesis better…”* (A2).

#### Social

Support from social or sport peers, the atmosphere on the team or the feeling of unity or being one with the team, increasing the number of social contacts and the presence of a sports partner were factors that were characteristic of this category.

#### Social group

Having the support and encouragement of others allowed individuals with LLA to feel important. *“I noticed that a lot of people from my community appreciate the fact that I sport….and the reactions that I receive really stimulate me…”* (A2). Their families or close friends are also important to constantly motivate and support their actions. *“My wife chases me out of the house.* <laughs> *… Now, that’s enough.”* (A9) or *“my partner supports me in everything I do.”* (NA11).

#### Sports partners

Increasing the number of social contacts or even the desire to be part of a group motivates individuals with LLA to participate in sports. Some mentioned that *“the social contacts are really important”* (A1) and that during sports you have the opportunity “…*to be part of a group…*” (A13). Taking part in group sports is *“fun”* (A9) and also gives the athlete the feeling of becoming “*one with the team”* (A13). Some individuals with LLA prefer to be part of a team in which teammates have a similar or somewhat equivalent degree of disability and this motivates them to participate in sports more frequently. *“It doesn’t matter how you do it because everybody has something, then you feel more at home and less stared at …… you feel less different….and then you accept it* <your disability>…*”* (A5). Non-athletes mentioned that if they would have a sports partner this would help them to start participating in sports*: “If I would have somebody, who will do the same thing……then you go more easily there* <sport>, *than alone.”* (NA5).

#### Personal

Factors and themes related to physical health or psychological attributes of individuals with LLA were included in this category. Additionally, themes represented by personal characteristics such as age and previous experience are also part of this category. It is worth mentioning that athletes mentioned a change in the facilitators to participate in sports before and after amputation. If prior to the amputation *“sport was never a priority, due to a rich social life and a busy schedule…” (A*1), it became more important following the amputation. This change in priority was often triggered by personal factors related to physical or psychological characteristics. In general it was observed that athletes were also active prior to their amputation *“Before my accident I used to ice-skate a lot and also to play football and to cycle……and this always leaves an imprint”* (A10).

#### Physical

Improving or maintaining physical health was the motivator to participate in sports mentioned by all 26 interviewees, including both athletes and non-athletes. The need to reduce the body weight or to increase physical fitness were two of the reasons most commonly identified during data analysis. *“I was really overweight, I had a bad physical condition. After 100 meters I began to feel tired, but that was no disadvantage, I found it more stimulating”* (A1) The second most commonly seen factor was pain. Even if pain was perceived as a barrier for sports by some athletes, for most pain represented a motivator to participate in sports because “…*pain disappeared in the moment I exercised enough.”* (A2) or possibly because they became aware of the fact that *“…if I do not exercise I will experience pain, more pain…”* (A5). An interesting finding is that the majority of the athletes who experienced (phantom) pain mentioned that *“<*it*> decreased in intensity or even completely disappeared”* (A10) as a consequence of participating in sports.

#### Psychological

Athletes and non-athletes alike considered participation in sports to be a *“really nice and fun activity to do…”* (A2 & NA9). Athletes were more enthusiastic in their responses, saying that they “love sport” or that they “really can’t live without it”. For the ones who stated that they cannot live without it, *“sport is more a necessity”* (A4) and, even if it was *“not perceived as a fun activity”* (A5), the individual still participated in sports because otherwise he or she had the feeling that it would have negative consequences for his or her health. “…*I feel that is compulsory…I have to go and do it* <sport>…” (A5). Participation in sports helped individuals to *“release part of the daily tension”* (A1) and to *“become more relaxed and strong* <psychologically>*”* (NA6). Competition, an element present in most of the sports, was valued by all athletes. This competition can be with others or with oneself, to show oneself that you are capable of participating, or just to establish one’s own limits and afterwards to try and *“push them <*own limits*>”* (A8). If you are *“…successful, then you feel good and really enjoy this* <sport>.*”* (A12).

## Discussion

This qualitative study showed that various Technical, Social and Personal factors can be both barriers and facilitators for participation in sports for individuals with LLA. While the most frequently mentioned barriers had either a technical or a psychological background, trivialization from others and a lack of predisposition for participation in sports appeared to be more difficult to overcome. Regardless, athletes were able to find a solution to their problems and therefore they overcame most of the barriers that they faced. Athletes focused either on the various advantages that regular participation in sports has for physical and psychosocial well-being, or they were more aware of the negative impact physical inactivity may have on health. Remarkable for this study is how phantom pain and prostheses appear to influence participation in sports. Athletes mentioned that participation in sports represented one of the most effective remedies for (phantom) pain whereas most of the non-athletes mentioned that even better prostheses would not motivate them to be more active. Therefore, programs aiming to encourage individuals with LLA to participate in sports should focus on providing personal counselling aimed at identifying and solving specific personal problems and to provide personally tailored sport advice.

Even if we assigned the identified themes into 3 distinct categories, an interaction between these categories was observed during data analysis. For example, a technical factor such as transportation may deter participation in group sports and therefore may motivate an athlete to become more active in sports that do not involve a team (individual). Therefore, transportation may indirectly influence both the number of social contacts and the effect of group competitiveness. This relationship may be positive, with the individuals able to identify solutions to their problems and becoming more active in their close surrounding, or negative, as others will become inactive as they give up looking for additional possibilities in their close surroundings. As can be observed from the above example, a motivator for one individual can represent a barrier for another.

### Technical

Being dependent on public transportation, inadequate sports facilities and insufficient information were viewed by the majority of interviewees as barriers, similar to findings in the available literature [6], [18], [35]. One remarkable finding of our study concerns the influence of the prosthesis on participation in sports. Our data suggests that the prosthesis may have a minor influence on participation in sports of individuals with LLA. Even if there were some individuals with LLA who mentioned that their prosthesis influences their participation in sports in a negative way, these individuals were all non-athletes and had either limited or no experience with their prosthesis during sports. Some of the non-athletes considered that they have “the best possible prosthesis”. This statement can be interpreted in two different ways; one, they consider that they will never get a better prosthesis (specialized sport prosthesis) than the one they have at the moment; and two, they are satisfied with their prosthesis and they don’t consider it as a barrier for participation in sports. These considering, the prosthesis and its influence on sports participation should be addressed during each individual assessment. In the existing literature, the prosthesis is described as one of the most important factors influencing physical functioning, locomotion, aesthetic appearance and social interaction of individuals with LLA [14], [36], [37]. Most of the athletes preferred to participate in wheelchair sports or other sports that generally placed less stress on their residual limbs, fact also similar to previous findings [38], [39]. All athletes mentioned that the choice to use or not use a prosthesis was entirely personal and was not influenced in any way by the technical characteristics of the prosthesis.

In summary, it seems that technical factors may more likely represent a barrier for sports than a motivator. Additionally, considering the fact that most individuals with LLA participate in sports without their prosthesis, it may be wise to pay special attention to other technical factors, such as transportation and inadequate facilities.

### Social

Similar to findings in the relevant literature, both athletes and non-athletes considered sports to be a social event, allowing them to come in contact and interact with individuals that they otherwise would not [12], [40], [41]. Considering that the number of social contacts decreased following amputation, sports may represent a means by which individuals with LLA connect with other individuals, either with or without LLA, to increase the number of social contacts and also to feel they are part of a group. Some individuals with LLA identified trivialization from others as one of the main reasons to stop participating in group sports, or even worse, to stop participating in sports completely. This aspect is not new, and almost all individuals with physical disabilities encounter this issue [42]. Overcoming this trivialization is therefore imperative for taking part in mixed-group sports [36]. All interviewees also mentioned the important role their family and friends plays in their choice to participate or not in sports. Therefore it may be so that the family may be able to help or at least may motivate them to regularly participate in sports.

In summary, interaction with others is important and may sometimes be the single- most important factor that influences participation in sports for individuals with LLA. Special attention should be directed towards providing adequate counselling during which individuals with LLA learn stigma management and strategies for how to deal with trivialization from others. Additionally, it may be useful to involve the individual’s family and friends in this entire process.

### Personal

Consistent with findings in the available literature, most of the non-athlete who did not have a medical contraindication for exercise mentioned that the main barrier they experience is their own attitude towards sports; either they do not want to exercise, are too lazy to get out of bed or they are not in the mood to exercise [6], [19]. The presence of injuries or poor health represented the most common barrier for sports mentioned by both athletes and non-athletes. Athletes believed that a poor health status would motivate them to be more active, and only a serious health condition would hinder their participation in sports. Non-athletes, however, observed no difference between various levels of physical health; they simply stated that poor health status would have a negative impact on their participation in sports. Remarkably, athletes mentioned that the presence of phantom pain is a strong motivator to participate in sports, mostly because they felt that phantom pain disappears with exercise. Non-athletes did not have this experience, and they relied almost entirely on pain medication or other therapies to reduce pain. Using sports as therapy for phantom pain is in agreement with recent findings, which state that a combination of mind-body therapies may be effective in reducing phantom pain temporarily or in the long term [43].

An individual’s own experiences and thoughts about participating in sports related to personal attributes such as fear of injury, feeling dependent, self-efficacy, and one’s own limits or mental attributes, including laziness or lack of disposition, appears to influence the participation in sports in individuals with LLA. While participating in sports, some individuals with LLA may realize that they are no longer able to achieve the same level of athletic performance as prior the amputation. Some individuals may accept this fact and try to constantly improve themselves through constant practice. Others may find it difficult to accept the impact their disability has on their sport performance and, in the more fortunate case, try to find an alternate sport where their disability may be less hindering their performance or either stop completely with sports. For the last category of individuals, before trying to motivate them to participate in sports, perhaps it may be wiser to decrease the burden of amputation by adequate coaching focusing on disability acceptance. One of the major differences between athletes and non-athletes can be observed in the problem-solving strategies each category adopts. Athletes appeared to be more proactive in searching for a solution and also appeared less discouraged by failing. This trait helped the individuals in the group not only in relation to their participation in sports but also in everyday life. Except for the individuals who experience barriers impossible to remove or overcome, such as an extremely poor physical state that makes it impossible to be physically active for more than 5 minutes at time, the process of choosing to participate or not participate in sports appears to be based on the assessment of risks and benefits associated with participation [44]. They stated that “choices involving gains are often risk averse and choices involving losses are often risk taking”. Translated to our research this may imply that individuals with LLA who are more aware of the risks (e.g., injuries, costs, problematic transport, etc.) than the gains (e.g., physical and psycho-social well-being) may be more likely to be non-athletes, while the individuals with LLA who are more aware of what they may lose (e.g., physical and psychosocial well-being) if they do not participate are more likely to be athletes. For example, individuals who experienced firsthand the negative impact of not participating in sports are the ones who perceived participation in sports as compulsory. Therefore, future campaigns for public awareness should focus more on the importance of sports and weigh the benefits of sports against the possible losses/risks.

In summary, if the major advantages of participation in sports are presented in an adequate manner it may allow non-athletes to overcome personal barriers and become athletes. Additionally, the influence of core beliefs should be taken into consideration during the first assessment or first contact with a rehabilitation specialist.

### Strengths and Limitations

To the best of our knowledge, this is the first qualitative study that aims to identify perceived barriers and facilitators for participation in sports in athletes and non-athletes with LLA. A systematic review [14] formed the framework of our research and it helped us to gather a vast but specific amount of data [45]. In addition, most of criteria of good qualitative research [46] were either met or addressed by the current research: 1) The topic of research is relevant and of interest for the professionals working with individuals with LLA and its results may help to increase the percentage of individuals with LLA that participate in sports; 2) Data gathered was analyzed by individuals with both clinical and theoretical experience; 3) All research steps are present in a transparent manner through the manuscript; 4) The results are accompanied by multiple participants quotes; 5) Transferability of the results was addressed, while known literature is used for comparison; 6) Considering that less is known about sports participation of individuals with LLA, more specific on the factors that promote or hinder it, the insight provided by this study has both practical and theoretical importance; 7) Local medical ethics committee assessed the research methodology and concluded specific approval was needed for this study and the regional specifics were considered when the semi-structured interview guide was constructed; 8) This study is coherent considering that the results and the methods of data gathering are in agreement with the aim of research.

Selection bias, given that we used only the database of a prosthetic manufacturer to recruit our interviewees, may represent a limitation to our research. Another possible limitation of our study is represented by the use of a rigid definition for sport. Some may argue that using our definition the individuals who are active 3 sessions per week maximum 29 minutes per session will be labelled as non-athletes while they may gather more weekly exercise time than athletes. Nevertheless, a theoretical cut-off point is needed in order to differentiate between athletes and non-athletes. Our definition intends to do merely this using a well-known and used parameter in the field of physical exercise. In general, athletes were younger, better-educated and had a more distal amputation (for reasons other than vascular disease) compared to non-athletes who were on average older, less educated and exhibited a more proximal amputation due to vascular reasons. Even so, neither groups considered these factors influential for participation in sports. Therefore, it may be that the differences in population characteristics between athletes and non-athletes did not represent a limitation for the current study.

### Conclusions

Programs aiming to promote participation in sports by individuals with LLA should first address the barriers and facilitators for participation in sports and only afterwards provide tailored advice that considers individual characteristics, such as sport desires, area capabilities, physical traits, psychological traits and previous experiences. Athletes appeared to be more proactive in searching for a solution and also appeared less discouraged by failing.

## Supporting Information

Appendix S1 **Interview guide used for interviewing athletes.**
(DOC)
