# Supplementary material for: Barriers and Facilitators of Participation in Sports: A Qualitative Study on Dutch Individuals with Lower Limb Amputation
Source: PLoS One. 2013 Mar 22;8(3):e59881. doi: 10.1371/journal.pone.0059881 (PMC3606215; doi:10.1371/journal.pone.0059881)
Supplement: Appendix S1 — Interview guide used for interviewing athletes. (DOC) [file pone.0059881.s001.doc]

**Appendix S1**

1. **Warming up / Informal conversation**
2. **Questionnaire with personal details**
3. **Semi-structured Interview**
   1. Why do you sport?
      - What is your reason to sport?
      - What could be a reason for you to stop participation in sport?
      - Did you sport also before amputation?
      - Is there a difference in the reasons to sport between pre and post amputation?
      - What do you see as advantages and disadvantages of sport?
      - Do you experience support during your regular participation in sports?
      - From whom **DO** you receive and from whom **DO you NOT** receive?
      - What motivates / demotivates you?
      - What situations did you experience, during your participation in sports, that were at first problematic but later you manage to overcome?
      - What situations did you experience, during your participation in sports, that were at first problematic and you did not manage to overcome them?

**Last question**

Do you want to add something / do you feel that we missed something, related to the topic of the interview?

**Checklist factors**

- Other disabilities
- Health (prefer to do other things / tired)
- Prosthesis
  - Yes / No prosthesis during sports?
  - Satisfaction related to prosthetist / prosthesis?
- Sport facilities?
- Information/advise
  - Received? Yes / No
  - Who do you consider to be the right person to provide information about sports and how?
- Time
  - Too much? / too less?
- Pain / phantom pain
- Awareness of own limits
  - + (increase?)
  - - (facing problems?)
- Fear
- Shame for others
- Dependence of others (e.g. transport/dressing)
  - Do you find it unpleasant to ask for help? Yes / No
- Age
- Previous negative experience
- Costs / income
- Sport companionship
  - + (e.g. yes, fun.)

- (e.g. nobody / no intention to group sport / unnecessary (alone/enough social contacts))
